# Attrition from Face-to-Face Pediatric Outpatient Chronic Pain Interventions: A Narrative Review and Theoretical Model

**DOI:** 10.3390/children11010126

**Published:** 2024-01-19

**Authors:** Kristen Tiong, Greta M. Palmer, Tiina Jaaniste

**Affiliations:** 1 School of Clinical Medicine, University of New South Wales, Kensington, NSW 2052, Australia; k.tiong@unsw.edu.au; 2 Department of Pain, Sydney Children’s Hospital, Randwick, NSW 2031, Australia; 3 Children’s Pain Management Service, Department of Anaesthesia and Pain Management, Royal Children’s Hospital, Melbourne, VIC 3052, Australia; greta.palmer@rch.org.au; 4 Department of Paediatrics, University of Melbourne, Melbourne, VIC 3052, Australia; 5 Murdoch Children’s Research Institute, Melbourne, VIC 3052, Australia

**Keywords:** attrition, chronic pain, pediatric

## Abstract

There is limited understanding of attrition (premature treatment withdrawal and non-completion) from pediatric chronic pain services. This narrative review aimed to summarize attrition prevalence from face-to-face pediatric outpatient chronic pain interventions, identify associated factors and develop a theoretical model to account for attrition in this setting. A comprehensive search of the published literature revealed massive variability (0–100%) in the reported attrition rates from pediatric chronic pain interventions that varied in type and format (individual vs. group, single discipline vs. interdisciplinary, psychological only vs. multiple combined interventions, of different durations). The factors associated with attrition from pediatric chronic pain programs varied between the studies: some have assessed patient sex, psychological and other comorbidities, avoidance strategies, missed schooling, family composition/tensions, caregiver catastrophizing, scheduling, caregiver leave and clinic access. A theoretical model is presented depicting youth, caregiver and service factors that may impact attrition from pediatric chronic pain interventions. Where available, literature is drawn from the pediatric chronic pain context, but also from adult chronic pain and pediatric weight management fields. The implications for research and clinical practice are discussed, including improved reporting, patient screening and targeted supports to promote intervention completion. This review contributes to a better understanding of attrition, which is crucial for optimizing pediatric chronic pain service outcomes.

## 1. Introduction

The term ‘attrition’ in the healthcare literature generally refers to the cessation or dropout of participants from a commenced study or intervention, but may also include loss to intended follow-up [1,2]. Within a clinical healthcare setting, attrition can be described as a patient withdrawing from a service before the clinician and patient agree that the intervention is complete [3,4]. These patients are also sometimes described as ‘non-completers’. Notably, some studies also use the term ‘non-completers’ or ‘partial completers’ to describe individuals who do not complete a minimum number of sessions [5,6]. Within a research context, the term ‘withdrawers’ is also sometimes used to describe participants for whom consent to proceed has been withdrawn, which can occur prior to or once having commenced the trial. The term ‘dropout’ has also been used [4,7], but arguably, ‘attrition’ has less negative connotations for the participant. There is the additional issue, generally considered separate from attrition, in which individuals have agreed or formally consented to participate with an intervention but who fail to start the intervention, i.e., ‘non-starters’.

The implications of patient attrition from a healthcare intervention are substantial. Attrition limits the healthcare benefits individuals receive, potentially amplifying health-related disparities [8]. Healthcare systems are also negatively affected by attrition, as the time and resources utilized are not effectively used to maximize patient outcomes. Moreover, in the context of healthcare research, patient attrition may compromise the validity of a study’s conclusions [9,10], particularly if withdrawal from treatment conditions is non-random [11].

Various face-to-face outpatient interventions are available for children and youth with chronic pain, including numerous approaches with well-documented efficacy [12,13,14,15,16]. Such interventions commonly involve several disciplines of health professionals, utilize physical, pharmacological, behavioral, psychological and social coping strategies [17,18], may require the involvement and support of parents/caregivers [19,20] and may take numerous weeks or months to complete. Despite established effectiveness, considerable numbers of patients who commence an interdisciplinary pain intervention withdraw prematurely, and thus do not experience potential improvements [14,21]. With the limited availability of pediatric chronic pain services, and many services having considerable waiting lists [22], attrition is likely to mean that resources are not optimally utilized.

Although most journals mandate that treatment studies give accurate and detailed reporting of patient enrolment and treatment completion rates [9,23], relatively little is known about attrition from face-to-face pediatric outpatient chronic pain interventions and factors associated with this attrition. No prior reviews of attrition in this clinical setting have been performed. A better understanding of the rates of, and reasons for, attrition from a service can be useful in determining where and how clinical efforts can be best directed. Hence, the aims of the current review were threefold. The first was to summarize the available attrition prevalence data within the pediatric chronic pain context, identifying the variability of definitions and reporting practices. The second was to identify patient, parent/caregiver and service factors found to be associated with patient attrition, identifying areas for future research. Given the limited work that has been carried out investigating attrition in the pediatric chronic pain context, the current review also draws on the attrition literature from other healthcare contexts, such as pediatric weight management programs and adult chronic pain programs. The third aim was to draw on the available literature to develop a theoretical model accounting for attrition from pediatric chronic pain services. Such a model may help identify different reasons for attrition that may occur at various stages of the treatment process.

## 2. Prevalence of Attrition from Pediatric Chronic Pain Interventions

A comprehensive, narrative review was conducted, using some systematic search strategies, to identify attrition rates from at least partially face-to-face pediatric outpatient chronic pain interventions. A search of the Medline(R) database was conducted for full-text English manuscripts published up to November 2023, using variants of the following search terms: pediatric, chronic pain, intervention and attrition. The references of these articles were also manually searched.

Table 1 summarizes 19 identified studies, reporting attrition or treatment non-completion from face-to-face pediatric outpatient chronic pain interventions. The interventions varied in terms of whether they were implemented by a single discipline or interdisciplinary team, group-based or individual, and importantly, the content of the session, including cognitive behavioral therapy (CBT), mindfulness-based interventions, stress and resilience training, yoga, as well as rehabilitation and pain management programs incorporating multiple components (sometimes with limited program-specific details). The intervention duration ranged from 4 sessions through to 16 sessions, and with some interventions having a variable number of sessions depending on need.

The way in which attrition was operationalized differed widely across the studies in Table 1, as shown in the right-most column. Attrition was commonly assessed as the percentage of patients who stopped attending the intervention before treatment completion (non-completers). However, some articles reported the proportion of patients who failed to attend (non-attenders) or defined treatment completers as attending a specified minimum number of sessions, for example, 2 out of 5 (40%; [32]), or 6 out of 8 (75%; [5,6]). Many studies defined or implied completion as full 100% attendance, but most did not provide detail regarding “partial completers” in their attrition figures. Most of the articles have considered attrition as drop-out following the commencement of the interventions, with some providing additional detail regarding those who were enrolled but did not proceed to intervention commencement (non-starters) [24,30,37]. The sample sizes ranged markedly, from less than 10 participants for some pilot studies (e.g., Jastrowski Mano et al., 2013; Lovas et al., 2017 [36,37]) through to over a thousand patients (Hechler et al., 2014 [28]). A number of smaller studies also included incentives for participation or questionnaire completion (e.g., Ali et al., 2017; Bakshi et al., 2021; Lee et al., 2023 [32,33,38]). The risk of bias was not formally analyzed as this was a narrative review and the majority of the studies were pilot studies with small samples.

Given the variability of the interventions and the definitions of attrition/non-completion, there is currently limited value in comparing attrition rates across all the studies. Suffice to say, that a considerable number of patients who commenced the interventions did not complete these. Some reasons for attrition provided by these studies were loss to follow-up [25,33,36], scheduling conflicts [26,27,34,35], transportation difficulties [27,34], required psychiatric treatment [26,27,35] and caregivers unable to get leave from work [24].

## 3. Evidence Regarding Factors Associated with Attrition from Pediatric Interventions and Adult Chronic Pain Interventions

The available evidence for how various (i) youth factors, (ii) caregiver/family factors and (iii) health service factors are associated with patient attrition are described below. Where available, evidence has been provided from the context of attrition from pediatric chronic pain interventions. However, where there is insufficient evidence, we have drawn from other health interventions, such as for pediatric weight loss and adult chronic pain.

### 3.1. Youth Factors

A small number of studies have compared the patient characteristics of youth who did versus did not complete a pain intervention [29,30]. The non-completers of a CBT intervention for chronic pain and anxiety did not differ significantly from treatment completers in age, sex, race, ethnicity, insurance status, anxiety status (subclinical versus clinical level) and pain intensity [30]. Carter et al. (2015) found that youth who dropped out of a manualized psychosocial intervention for chronic painful and fatiguing conditions were reported as more likely to be male, to use avoidance strategies, and make less use of active problem-solving strategies or humor relative to treatment completers, with no racial differences [29]. Furthermore, patients who dropped out of the intervention had more comorbid unexplained painful and fatiguing conditions and were more likely to have repeated a school grade than treatment completers. Similarly, research with adult chronic pain patients has found lower educational attainment to be associated with treatment attrition [40].

No pediatric chronic pain studies have assessed patient age in association with attrition. In pediatric weight management studies, older age is a significant predictor of attrition [41,42]. This may potentially be because adolescence is associated with increasing independence and decreasing caregiver authority in health-related matters [43]. A systematic review (including eight studies) investigating factors associated with attrition from adult interdisciplinary pain management programs reported that higher levels of pain intensity and disability were predictors of attrition, although some conflicting results were acknowledged [4]. Moreover, in the adult pain context, a longer duration of disability has been found to be associated with greater attrition [44,45]. However, the associations between pain intensity, disability, pain duration and attrition have not been examined in the context of pediatric chronic pain interventions.

It has been well documented in the context of pediatric weight management programs that unrealistic or unaddressed patient program expectations are associated with treatment attrition [41,46,47]. We have found no data regarding the relationship between patient expectations and attrition from pediatric chronic pain interventions. However, as negative beliefs and attitudes regarding a pain intervention are strongly associated with low adherence to treatment recommendations [48], treatment attrition is likely to follow.

Within the context of adult chronic pain interventions, attrition has been found to be associated with catastrophizing [49,50], lower self-efficacy [49] and low readiness for change [51]. Further, in the context of pediatric weight management interventions, depressive symptoms [42], lower self-concept [42], and bullying [52] were associated with greater attrition. These factors are yet to be considered in the context of attrition from pediatric chronic pain interventions.

### 3.2. Caregiver and Family Factors

Caregivers have an instrumental role in facilitating the attendance of children and youth at pediatric chronic pain services, with children typically reliant on caregivers for diarizing appointments and arranging transport. Caregivers also have an important role in facilitating treatment engagement and adherence to recommendations, typically as key stakeholders in decision-making pertaining to continuation or attrition from an intervention. In light of the significant levels of burden experienced by caregivers of children with a chronic pain condition [53,54,55], it is important to consider their capacity for facilitating their child’s attendance at a chronic pain service.

Caregiver and family factors have not been well examined in relation to attrition from pediatric chronic pain services. One study has compared parent and family factors for youth who stopped attending a pediatric chronic pain intervention with those who completed treatment [29]. No significant differences were found in the family composition (single-parent vs. two parent), marital status, or presence of family tension.

Lower educational attainment and unemployment is associated with attrition from adult chronic pain interventions [40,56], and may be relevant for caregivers of pediatric chronic pain patients. Caregivers with low educational attainment or low health literacy may have difficulty following explanations and treatment rationales, potentially contributing to attrition. This is of salience given that the prevalence of low health literacy among adults is 47% in European samples [57], 60% in Australian adults [58], and between 28% [59,60] and 70% [61,62] in North American parents. This low health literacy potentially impacts caregivers’ ability to utilize health information in making informed decisions for their child [58].

Within the pediatric chronic pain context, the role of caregiver beliefs and attitudes has not been well studied in so far as it may impact patient attrition. Higher caregiver catastrophizing has been found to be significantly associated with greater maladaptive caregiver behaviors (such as being overprotective or oversolicitous), increased child pain intensity and disability [63] and the non-acceptance of the evidence-based functional approach of interdisciplinary pain interventions [63,64]. However, it is not known whether caregiver catastrophizing is associated with attrition from a pediatric chronic pain service.

Parental and youth expectations have not been studied in the context of attrition from pediatric chronic pain interventions. In the context of pediatric weight management programs, one study documented that 37% of the parents of patients who dropped out reported the program did not meet their expectations [65].

### 3.3. Health Service Factors

Very little research has been conducted investigating the role of health service factors on patient attrition from pediatric interventions. For interdisciplinary pediatric weight management interventions, health service factors that have been found to be related to patient attrition include intervention complexity [41,66], time commitment [41,67], inadequate or excessive frequency of appointments [41,65], and program dissatisfaction [65,67]. Transportation difficulties [52] and the distance to services [47,65,67] are also noted barriers to ongoing attendance.

## 4. Developing a Theoretical Model for Understanding Patient Attrition

A valuable theoretical model for understanding treatment attrition has been developed in the context of adult psychotherapy [3]. This model focused on the early therapeutic environment in which patient attrition is related to three concepts, namely (1) failure to achieve a holistic connection between the client and therapist, (2) client distress that remains too high or too low for effective engagement, and (3) low client efficacy and outcome expectations regarding therapy and related behaviors.

A model for understanding attrition from a pediatric health intervention (such as an outpatient chronic pain program) is proposed, building on some aspects of the above model. Importantly, a model of attrition from pediatric pain interventions must incorporate the complex interplay between caregiver and patient factors. This includes not only factors that occur within the early therapeutic environment, but also the patient’s and caregiver’s histories in so much as their past experiences and beliefs may impact on their actions and engagement.

**Figure 1:**
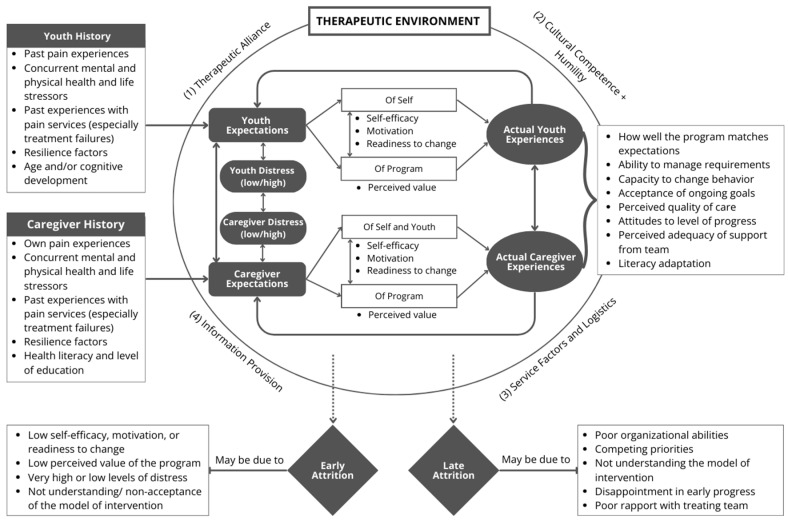
Model of patient attrition from pediatric outpatient chronic pain interventions.

Figure 1 depicts a number of broad concepts that may relate to attrition from a pediatric pain service, namely (i) the therapeutic environment created by the health service and treating team, (ii) the expectations and distress levels of the caregiver and youth, upon commencing the intervention and thereafter, (iii) the actual experiences of both the caregiver and youth of the program, and (iv) background caregiver and youth factors that may shape their expectations, behaviors and experience of the program. Each of these aspects will be considered below in more detail, noting how certain factors may be more likely to contribute to either early or later attrition. Of particular note is the fact that at each level of this model, there may be complex interactions, as indicated diagrammatically, including the bi-directional interplay in Figure 1, between caregivers and patients, potentially contributing to attrition risk.

### 4.1. The Therapeutic Environment

Healthcare interventions, such as pediatric chronic pain programs, are delivered within the broader context of a therapeutic or treatment environment. While a positive therapeutic environment is generally insufficient to lead to positive treatment outcomes (requiring efficacious interventions), a negative therapeutic environment may well contribute to treatment failure and/or attrition. Our model of attrition from pediatric outpatient chronic pain services addresses four key aspects of the therapeutic environment.

(1) The therapeutic alliance or rapport established between health professionals and patients/caregivers is well recognized as important in the psychotherapy context [3,68,69,70]. In various adult healthcare contexts, the patient–therapist relationship has been shown to impact treatment outcomes [71], with mixed results in the context of adult chronic pain [72]. A discussion of possible mechanisms by which therapeutic alliance drives therapeutic change has been outlined recently [68], and is beyond the scope of the current review paper. A positive patient–therapist relationship may help to motivate and engage patients with the treatment components, resulting in a lower likelihood of attrition. Moreover, a good rapport between the therapist and youth/caregiver enables the therapist to clarify and impress the rationale for the proposed intervention, minimizing any negative preconceptions [29]. Further, therapists who have a good rapport with the family are in a better position to be able to explore the patient’s and caregiver’s beliefs about the pain condition and the proposed management approach to prevent expectation mismatch [73,74]. Doing so may minimize dissatisfaction and potentially reduce the risk of attrition.

(2) Cultural competence refers to familiarization with the cultural beliefs and practices of specific cultures, particularly related to healthcare [68,75,76,77]. Cultural humility, on the other hand, recognizes that it is not always possible for clinicians to be knowledgeable and competent in all cultures, but rather typifies a mindset with acceptance of difference and that welcomes diversity [78,79]. Cultural competence and/or cultural humility are of importance in creating a ‘safe space’ in which a healthcare intervention can occur. If these are absent, then this will likely lead to a lack of understanding and failure to bridge discrepancies between the family’s underlying healthcare assumptions relative to the dominant healthcare culture and may result in attrition from healthcare interventions [80,81]. There is limited research exploring the treatment experience and attrition rates among pediatric chronic pain patients with culturally diverse backgrounds. However, if instructions and rationale are not clearly understood, engagement and adherence with treatment requirements are less likely to be achieved, and attrition may result.

(3) A range of organizational and service factors may contribute to the therapeutic context in which an intervention can occur. Examples that can impact on the patient’s or family’s experience and ease of engagement include the navigation of logistical issues (such as parking, transport access and options, travel distance, appointment bookings, access to interpreters), as well as the physical environment in which the appointments take place (including age-appropriate furnishings). If practical challenges associated with attending a service exceed the family’s coping resources, attrition may be more likely to occur.

(4) The way in which information provision occurs contributes to the context in which an intervention can take place. Effective communication and information provision to both the patient and the caregiver helps maximize accurate patient and caregiver expectations [82], thereby avoiding frustrations and misunderstandings that can lead to attrition. The way in which key concepts and treatment models are explained to patients and caregivers may contribute to the way in which they engage with an intervention [83]. The capacity for the treating team to adapt the program for varying literacy levels is also of likely importance.

### 4.2. Expectations and Distress Levels of the Youth and Caregiver

Caregivers and youth have expectations of themselves, and their ability to meet treatment requirements, as well as expectations of a treatment program. Individuals who do not believe that they have the capacity to meet treatment requirements may opt to withdraw from treatment. Likewise, if they do not expect an intervention to be of value to them, they are more likely to cease attendance. These expectations are likely to be formulated following early appointments with the service, where the nature of the treatment and treatment expectations are explained to the family. Moreover, caregiver and patient expectations may shape each other. For example, if caregivers hold low expectations of a program, they are less likely to encourage the patient to engage with the intervention in a positive way, thus potentially lowering the patient’s own expectations and prospects of success.

It has been argued in the classic paper by Bandura (1989), that belief or expectation in one’s ability to exercise agency in a situation will influence motivational, cognitive and affective processes, and ultimately behavior [84]. Similarly, the concept of ‘readiness to change’ relates to an individual’s preparedness to make physical, psychological or social changes that an intervention may require [85]. Others have suggested that an individual’s expectations and potentially their readiness to change may be modified through techniques such as motivational interviewing [86]. The expectations of a child/youth and their caregiver may influence each other, although differences may lead to tension and conflict, particularly when either or both are not aligned with the therapist’s treatment goals for the patient.

Youth and caregiver distress upon commencing an intervention may influence each other and, if not addressed promptly, may impact the ability to engage with an intervention. It has been proposed by Meier et al. (2022) [3], that individuals with very high or very low distress may be more likely to drop out of a therapeutic intervention. Patients with high distress levels may be overwhelmed and lack the capacity to engage with treatment requirements. Conversely, patients with low distress levels may lack the motivation or drive to engage and implement treatment recommendations [3].

### 4.3. Actual Experiences of Youth and Caregivers of the Intervention

After engaging with a treatment intervention for a period of time, patients and caregivers may consider the extent to which the intervention matches their expectations. Patients and families will consider their level of satisfaction with the service and their own ability to meet treatment requirements. Attrition may result if the patient and/or caregiver have found it difficult to commit to the treatment requirements, perhaps due to competing priorities or other stressors. Further, if the family is dissatisfied with the level of progress that has been achieved since commencing the intervention, attrition may also occur. The differences between the model’s proposed reasons for early and late attrition, as shown in Figure 1, extend on work in the adult pain context [40,87]. Early attrition may be due to low expectations of themselves or the program or not understanding nor accepting the model, which affects engagement with the intervention. Late attrition may be related to external factors that prevent attendance despite a desire to continue, or issues with adequate rapport, meeting expectations or the misunderstanding of the model of the intervention.

### 4.4. Background Youth/Caregiver Factors

Finally, youth and caregivers enter the treatment context with a broad range of factors that may enhance or reduce their likelihood of completing treatment. It has been found in various other contexts that patients who have had past treatment failures are less likely to succeed with subsequent interventions [88,89], finding it harder to engage [88]. Moreover, pediatric chronic pain patients or their caregivers who have comorbid mental health conditions, comorbid physical conditions or other life stressors may find it more difficult to meet treatment requirements [29]. However, resilience factors such as positive coping styles, flexibility and an openness to the role of psychological factors, as well as good social supports, may render patients and families in a better position to engage with treatment requirements [90,91,92]. Patient age is also included in the model, given that attrition may be more likely among older adolescents who may have greater decision-making capacity independent of their parents [93]. As chronic pain commonly occurs within families [94,95], the caregiver’s experience with their own or other family members’ chronic pain, and the type of interventions tried, may impact on how the family engages with the current intervention. Little is known about whether the level of patient or caregiver education impacts on attrition, although some evidence of this has been found in other pediatric lifestyle interventions [96]. In the context of pediatric chronic pain interventions, it is possible that a higher level of education helps families better grasp biopsychosocial and neurobiological explanations and frameworks that are commonly presented as part of the pain education and intervention.

## 5. Limitations and Future Research and Clinical Directions

As outlined in Section 2, varying definitions of attrition, as well as very different interventions targeting pediatric chronic pain, make drawing comparisons regarding patient attrition across studies difficult. There is variability in the amount of information reported in manuscripts regarding attrition from interventions, with details such as timing and reasons for attrition not always reported. Moreover, attrition data generally do not incorporate the considerable number of additional patients who are referred to, or enrolled in, an intervention but who do not attend the initial assessment [97]. A greater uniformity of attrition definitions is necessary and reporting should include details of the time points and reasons of attrition. This would assist in the development of strategies to reduce barriers to effective interventions, optimizing engagement and outcomes.

Differences also need to be acknowledged between attrition data reported from research and clinical settings, with relatively little attrition data available from routine clinical settings. Unlike research-based interventions, which are more commonly manualized and highly standardized, clinical practice offers greater scope for the tailoring of interventions and greater flexibility in appointment spacing to better align with a family’s other commitments. Clinicians may persist with attempting to engage families even after multiple non-attendances (which might disqualify participants from a study protocol). Moreover, there is typically some participant self-selection in research studies [98,99], and sometimes the use of participation incentives [100,101], both of which may potentially impact on compliance and attrition, especially when incentives are as large as USD 200, such as the study by Ali et al. (2017) [33]. Finally, care should be taken when interpreting attrition rates from studies with very small sample sizes, as large studies will afford more robust data.

Although the current review benefits from drawing on contexts other than pediatric chronic pain, it should be acknowledged that there may be differing factors related to attrition in other pediatric interventions (such as for weight management) and adult chronic pain interventions. For example, the psychosocial factors may differ for a condition that is visible, such as obesity, relative to an invisible condition, such as chronic pain. Attrition from adult chronic pain interventions would also differ in that adult patients may or may not have caregivers with an instrumental role in their attendance and engagement. There are also differing costs and potential secondary gains associated with an unsuccessful treatment, regarding school attendance and engagement for youth, relative to the ability of adult patients or youth to maintain, regain or gain future employment.

Although the current review focused on attrition from face-to-face, pediatric outpatient chronic pain interventions, there is also a need to consider the concept of attrition from remote interventions in this field. A systematic review of remote psychological interventions in the context of children’s chronic or recurrent pain found that seven of 10 studies provided unclear or incomplete information regarding attrition [17,102]. They failed to report attrition data, reasons for attrition, or any assessment of the difference between completers and non-completers. More work is needed in this area, starting with improved clarity on how attrition from a remote intervention may be best defined.

A stronger evidence base regarding factors associated with attrition from pediatric chronic pain services may permit the determination of and focus upon patients at high risk of not completing an intervention. This has been performed in the context of an adult lifestyle intervention, where knowledge of attrition factors has been used for the development of a screening tool identifying patients at the greatest risk of attrition with fair to moderate discriminatory power [103]. Some services may choose to consider whether commencing an intervention with a patient at high risk of attrition is a good use of finite resources, potentially also resulting in a failure experience for the patient. Alternatively, consideration could be given to whether attrition risk factors can be addressed and minimized through tailored, patient-centered care, utilizing appropriate retention strategies. General attendance strategies have been described in the literature, including various types of appointment reminders [104,105] and pre-appointment surveys [106]; however, these are yet to be evaluated in terms of their potential impact on attrition from pediatric chronic pain interventions. Consideration is also needed as to whether different retention strategies are most suited to different modalities of intervention, with potentially different factors contributing to attrition from individual versus group-based programs. Furthermore, clinical teams would benefit from the explicit consideration of strategies to optimize the therapeutic alliance, as well as organizational and service factors. Finally, evaluating a patient’s readiness for change [107] may allow for the tailoring of the approach to address pre-intervention barriers, which could improve patient engagement and the effectiveness of a service [108]. 

## 6. Conclusions

Highly variable attrition rates have been reported for face-to-face outpatient interventions targeting pediatric chronic pain, largely due to the differing interventions offered, and the different methods used to operationalize attrition. The current narrative review identified a range of youth, caregiver, and health service factors with some evidence to suggest an association with patient attrition from outpatient health interventions. Further research and an audit of clinical services in the pediatric chronic pain intervention context should examine factors within these domains and ascertain clinical attrition rates (including the timing of and reasons for) to understand the impact of attrition. This is necessary to highlight areas for improvement in optimizing the engagement of youth and families at risk of attrition. It is a critical step to developing tailored, patient-centered, and best-practice clinical care that minimizes attrition and maximizes outcomes within these beneficial, evidence-based interventions. Finally, the theoretical model of attrition from outpatient pediatric chronic pain services that has been offered in the current manuscript seeks to promote further research and the clinical consideration of relevant factors related to attrition from pediatric chronic pain interventions.

## Figures and Tables

**Table 1:** Attrition rates for outpatient, face-to-face pediatric chronic pain interventions.

**First Author** **Year** **Country** **Study Type**	**Sample n**	**Age, Years** **(% Females [F])**	**Chronic Pain Conditions**	**Intervention**	**Delivery**	**Intervention Duration**	**Attrition** % of n *	**Definition of Attrition and/or Non-Completion Used**
**Interdisciplinary with Physical Therapy [PT] component**								
Hilyard et al., 2020 [24] Australia Pilot	(i) 6 families (ii) 5 families	12–17 (91% F)	Chronic pain	(i) Hybrid (F2F and VC) ped. I-D pain program (clin. psychol., PT and OT) (ii) F2F sessions (standard care)	(i) Group F2F and VC, and Indiv. VC [Hybrid] (ii) Ind. F2F only [SC]	7 weeks with 14.5 h total 11 h group; 3.5 h Indiv.	(i) 17% withdrew before starting drug trial (ii) 17% withdrew before starting SC 1/5, 20% loss to FU/declined questionnaires	Lost to FU OR declined questionnaires after starting intervention
Ocay et al., 2023 [25] Canada Cohort	414	8–18 (83% F)	Chronic pain	Personalized I-D treatment (pharm., PT, psychol., nursing, SW and interventional)	Indiv. F2F	Variable (not specified)	7.9% overall (i) loss to FU 3.1%; (ii) dropout 4.8%	Lost to FU OR dropout due to non-compliance with planned treatment.
Shear et al., 2022 [26] USA RCT	68	M = 14.2 (81% F)	Chronic pain	(i) I-D graded exposure treatment (ii) M-D pain management Joint delivery psychol. and PT	Mixture: F2F, VC and Hybrid.	12 × 1 h (twice weekly) 6 weeks 12 h	13% (3/9 withdrew related to COVID-19 reasons)	Withdrew from treatment before completion
Tran et al., 2017 [27] USA Pilot	30	12–18 (100% F)	Juvenile fibromyalgia	CBT/exercise. Joint psychol. postdoctoral fellow/ped. pain psychol. and EP/PT	Group F2F	2 × 1.5 h 8 weeks 24 h	Dropouts 20%	Dropout after starting program
**Interdisciplinary without Physical Therapy component**								
Hechler et al., 2011 [14] Germany Cohort	275	4–18 (56% F)	Chronic Pain	Multimodal treatment with pharm. and psychol. components	Indiv. F2F	Variable	Dropouts 18%, 12%, 16% at 3, 6, 12 months Non-attendants (non-completers) 8%, 24%, 32% at 3, 6, 12 months	Study dropouts: withdrew entirely from study Non-attendants: discontinued multimodal treatment (non-completers)
Hechler et al., 2014 [28], Germany Cohort	1520	1–19 (58% F)	Chronic pain	I-D outpatient medical, and psychological treatment recommendations	Indiv. F2F	Variable	35% did not attend the required return visit	No return visit to treatment center within 12 months
**Cognitive Behavioral Therapy [CBT]**								
Carter et al., 2015 [29], USA Cohort	62	M = 14.6, SD = 1.6 (92.8% F)	Chronic painful and/or fatiguing conditions	Manualized psychosocial treatment: CBT, behavioral family systems therapy and interpersonal psychotherapy. Postdoctoral fellows, predoctoral interns, and ped. psychol. doctoral students	Indiv. F2F	12 × 1 h sessions 12 weeks 12 h	Dropouts 32%	Completers attended 12/12 sessions
Cunningham et al., 2016 [30], USA Cohort	175	8–18 (78% F)	Chronic pain and anxiety	Pain-focused CBT by clin. psychol. (specialized in ped. chronic pain or in advanced training)	Indiv. F2F	Variable (not specified)	Dropouts 44/84, 52% (52% non-starters)	Dropped out after intervention commenced
Kashikar-Zuck et al., 2012 [31], USA RCT	114	11–18 (92% F)	Juvenile fibromyalgia	CBT. 5 postdoctoral ped. psychol. and CBT-trained therapists	Indiv. F2F	8 × 45 min sessions 8 weeks 6 h	Dropouts 12/100, 12% (1.8% Non-starters)	Non-completion of treatment and follow-up
Lee et al., 2023 [32], USA Canada Pilot	72	Parents of 8–17 year olds (74% F)	Chronic Pain	Parent-targeted group CBT led by 2 facilitators (at least one clin. psychol. or clin. psychol. doctoral trainee)	Parents’ group F2F or VC	5 × 2 h 5 weeks 10 h (USD 5 gift card per questionnaire $15 for 3—pre, post, f/u)	5.6% overall F2F 1/27, 3.7%	Those who attended < 2 of the 5 sessions
**Mindfulness-Based Interventions [MBI]**								
Ali et al., 2017 [33], USA Cohort	18	10–18 (73% F)	Functional somatic syndromes	Mindfulness-Based Stress Reduction (MBSR) led by an experienced instructor	Group F2F	8 × 1.5 h, 8 weeks (plus 4 h retreat) 16 h ($200 USD for participation)	Dropouts 7%	Withdrew before completing all sessions
Chadi et al., 2016 [34], Canada Randomized pilot RCT	20	13–17 (100% F)	Chronic pain	MBI by two psychiatry residents	Group F2F	8 × 1.5 h sessions 8 weeks 12 h	Dropouts 5% (3 withdrew before randomization)	Non-completion of all 8 sessions
Hesse et al., 2015 [35], USA Pilot	20	11–16 (100% F)	Recurrent headaches	MBI by three MBSR trained instructors; clin. psychol. and psychiatrist present during all sessions	Group F2F	8 × 2 h evening sessions 8 weeks 16 h	Dropouts 25%	Of those enrolled, % that dropped out of intervention.
Jastrowski Mano et al., 2013 [36] USA Pilot	6	12–17 (75% F)	Chronic pain	MBSR delivered by experienced MBSR practitioner	Group F2F	6 × 1.5 h sessions 6 weeks 9 h	Psycho-education: lost to FU 2/2, 100% MBSR: lost to FU 2/4, 50%	Lost to FU at 4 weeks
Lovas et al., 2017 [37] Canada Pilot	18	14–17 (86% F)	Chronic pain	MBI taught by a child and adolescent psychiatrist with formal training in MBSR and MBCT	Group F2F	8 × 1.5 h sessions 8 weeks 12 h	Dropouts 0% of the 7 who commenced (61% Non-starters)	Completion of intervention by those who started
Ruskin et al., 2017 [6], Canada Pilot	21	12–18 (95% F)	Chronic pain	MBI for adolescents led by two facilitators completing MBI training	Group F2F	8 × 2 h (after school) 8 weeks 16 h	Non-completers 9.5% (0% Non-starters)	“Treatment completers” completed at least 6/8 sessions
Suc et al., 2022 [5], France Pilot	27	12–17 (67% F)	Chronic pain	MBI led by at least two experienced MBSR instructors: focus on building skills and incorporating meditation, exercises, and activities adapted for teenagers	Group F2F	8 × 1.5 h (after school) 8 weeks 12 h	Dropouts 11%	“Treatment completers” attended at least 6/8 sessions.
**Other**								
Bakshi et al., 2021 [38], USA Pilot	Part B: 9	15–18 (53% F)	Sickle-cell disease with chronic pain	Instructor-led yoga sessions	Group F2F	8 × 1 h 8 weeks 8 h (USD 25 for surveys completed, USD 25 for interviews, USD 1/diary entry, USD 25/yoga session)	89% did not complete any sessions One partial completer 11% 3/8 sessions	Not attending any of the 8 sessions despite enrollment
Gmuca et al., 2022 [39], USA Pilot	27	12–17 (82% F)	Musculo-skeletal chronic illness	Promoting Resilience in Stress Management (PRISM) coaching. Delivered by trained, bachelor-level coaches	Indiv Choice of F2F or VC telephone.	4 × 30–50 min over 3 months (1–2 weeks apart)	Dropouts 15%	Non-completion of each of the 4 required sessions by enrolled participants.

* Where the participant number is less than the initial sample size, the absolute numbers are provided in addition to the percentage. Legend: Clin. psychol., clinical psychologist; EP, exercise physiologist; F2F, face-to-face; F, female; FU, follow-up; I-D, interdisciplinary; Indiv., individual; M, mean; MBCT, mindfulness-based cognitive therapy; MBI, mindfulness-based intervention; MBSR, mindfulness-based stress reduction; M-D, multidisciplinary; OT, occupational therapist; Ped., pediatric; Pharm., pharmacology; Psychol., psychology; PT, physical therapy; RCT, randomized controlled trial; SD, standard deviation; SW, social work; VC, videoconference/virtual.

## Data Availability

Not applicable.
